# Co-Amorphous Formulations of Furosemide with Arginine and P-Glycoprotein Inhibitor Drugs

**DOI:** 10.3390/pharmaceutics13020171

**Published:** 2021-01-27

**Authors:** Marika Ruponen, Konsta Kettunen, Monica Santiago Pires, Riikka Laitinen

**Affiliations:** 1 School of Pharmacy, University of Eastern Finland, P.O. Box 1627, 70211 Kuopio, Finland; marika.ruponen@uef.fi (M.R.); konke@student.uef.fi (K.K.); 2015177@alunos.estesl.ipl.pt (M.S.P.); 2 Escola Superior de Tecnologia da Saúde de Lisboa, 1990-094 Lisbon, Portugal

**Keywords:** co-amorphous, dissolution, solubility, stability, permeation, P-gp inhibitor

## Abstract

In this study, the amino acid arginine (ARG) and P-glycoprotein (P-gp) inhibitors verapamil hydrochloride (VER), piperine (PIP) and quercetin (QRT) were used as co-formers for co-amorphous mixtures of a Biopharmaceutics classification system (BCS) class IV drug, furosemide (FUR). FUR mixtures with VER, PIP and QRT were prepared by solvent evaporation, and mixtures with ARG were prepared by spray drying in 1:1 and 1:2 molar ratios. The solid-state properties of the mixtures were characterized with X-ray powder diffraction (XRPD), Fourier-transform infrared spectroscopy (FTIR) and differential scanning calorimetry (DSC) in stability studies under different storage conditions. Simultaneous dissolution/permeation studies were conducted in side-by-side diffusion cells with a PAMPA (parallel artificial membrane permeability assay) membrane as a permeation barrier. It was observed with XRPD that ARG, VER and PIP formed co-amorphous mixtures with FUR at both molar ratios. DSC and FTIR revealed single glass transition values for the mixtures (except for FUR:VER 1:2), with the formation of intermolecular interactions between the components, especially salt formation between FUR and ARG. The co-amorphous mixtures were found to be stable for at least two months under an elevated temperature/humidity, except FUR:ARG 1:2, which was sensitive to humidity. The dissolution/permeation studies showed that only the co-amorphous FUR:ARG mixtures were able to enhance both the dissolution and permeation of FUR. Thus, it is concluded that formulating co-amorphous salts with ARG may be a promising option for poorly soluble/permeable FUR.

## 1. Introduction

During drug development, molecules categorized under the Biopharmaceutics classification system (BCS) as class IV, i.e., those compounds that show both insufficient solubility and problems in membrane permeability and/or active cellular efflux across intestinal membranes, are the most difficult from the oral formulation point of view [1]. The current “best” solution to improve the oral bioavailability of class IV drug compounds is to go back to the drug discovery phase and lead optimisation by modifying the molecular structures in order to obtain a compound with more appropriate physicochemical properties; however, this is both expensive and time consuming [1]. Therefore, in this study, we aimed to address these challenges from the point of view of formulation in a more widely applicable way, i.e., by synergistically combining solubility/dissolution enhancement with permeation enhancement by adopting a co-amorphous approach to promoting the bioavailability of the most difficult-to-deliver compounds, i.e., class IV drugs.

Co-amorphous systems are combinations of two or more low-molecular-weight components that form homogeneous, amorphous single-phase systems [2,3]. Co-amorphous combinations of two active drug compounds have been considered to represent interesting candidates for combination therapy [4,5,6,7]. Instead, mixtures of an active molecule and an excipient (e.g., an amino acid) can offer more general formulation options [8,9]. By both approaches, enhanced dissolution properties for poorly soluble drugs, as compared to their crystalline counterparts or individual amorphous forms, and the stabilization of an amorphous form, often by intermolecular interactions, have been achieved [10,11,12,13]. In addition, the abilities of drugs to permeate in co-amorphous combinations across synthetic PAMPA (parallel artificial membrane permeability assay) or cellular barriers have been found to improve due to enhanced dissolution [14,15,16].

In this study, we used furosemide (FUR) as a model drug. FUR is a problematic drug, as it belongs to BCS class IV but also with respect to preparation methods; it experiences thermal decomposition upon melting (a problem for melting methods) [17], it is poorly water soluble (problems with evaporation methods) [18], and it is easily degradable upon milling, even in the presence of excipients [19,20]. In spite of this, FUR has been claimed previously to be transformed into an amorphous form, and it has been claimed that co-amorphous formulations with amino acids could be successfully prepared by milling [21]. However, it has been found that the cryomilling of FUR alone or its binary mixtures with excipients, such as inulin or polyvinylpyrrolidone (PVP), leads to significant degradation due to hydrogen bonding [19]. Instead, when the process was conducted with acylated sugars, degradation was avoided, as an unusual hydrogen bond C−H−O by the furan group in furosemide with one of the numerous acetyl substituents in acylated glucose was formed [20]. Thus, we aimed at overcoming these stability issues and solubility problems by combination with excipients that would achieve sufficient solubility for FUR, enabling spray drying from aqueous solutions (arginine (ARG)). Furthermore, since it is evident that FUR is a substrate for efflux [18], we aimed at using known the P-glycoprotein (P-gp) inhibitors piperine (PIP), verapamil hydrochloride (VER) and quercetin (QRT) [1,22] as co-formers in co-amorphous mixtures prepared by evaporation from organic solutions. The co-amorphous mixtures were prepared at 1:1 and 1:2 drug:excipient ratios and characterized with respect to their solid-state properties (by X-ray diffraction (XRPD), differential scanning calorimetry (DSC) and Fourier-transform infrared spectroscopy (FTIR)) and physical stability. Simultaneous dissolution/permeation testing was performed to evaluate the enhancement of the dissolution and permeation properties of the formulations to increase the bioavailability of FUR.

## 2. Materials and Methods

### 2.1. Preparation of the Amorphous Materials

Furosemide (FUR, M = 330.745 g/mol, ≥97%; ThermoFisher GmbG, Kandel, Germany) was used as a model drug in combination with the co-formers l-arginine (ARG, M = 174.2 g/mol, ≥98%; Sigma-Aldrich, St. Louis, MO, USA), piperine (PIP, M = 285.34 g/mol; ≥98%, Cayman Chemical Company, Ann Arbor, MI, USA), quercetin (QRT, M = 302.24 g/mol, ≥98%; Sigma-Aldrich, St. Louis, MO, USA) and verapamil hydrochloride (VER, M = 454.6 g/mol; ≥98%, Cayman Chemical Company, Ann Arbor, MI, USA).

In the preliminary studies, the co-amorphous mixtures were prepared in situ in a differential scanning calorimeter (TA DCS 2500, TA Instruments, New Castle, DE, USA), with a RCS90 cooler (TA refrigerated cooling system 90, TA Instruments, New Castle, DE, USA) and nitrogen flow of 50 mL/min from physical mixtures (PMs). The PMs were prepared in an oscillatory mill (Mixer Mill MM400, Retsch GmbH&Co., Haan, Germany). The drug and a conformer with a total mass of 500 mg were transferred into 25 mL milling jars. Mixing was performed at 30 Hz for 5 min. The mixtures were stored at ambient temperature and humidity. Samples were weighed (Sartorius SE2, Sartorius AG, Goettingen, Germany) into hermetic aluminium pans pierced with a pin (TA Instruments, New Castle, DE, USA). The samples were equilibrated at 40 °C and heated at 10 °C/min until the melting points were reached (FUR–VER, 170 °C; FUR–PIP 1:1, 180 °C, and 1:2, 170 °C; FUR–ARG, 180 °C; and FUR–QRT, 215 °C). The temperature was held for 5 min at melting, after which fast cooling to −50 °C was performed. After this, the samples were heated at 10 °C/min to 215 °C (except FUR–QRT, heated to 320 °C). The results were obtained using TRIOS Software (TRIOS, TA Instruments, New Castle, DE, USA). The co-melting of FUR–PIP and FUR–VER was also attempted on a hot plate (Heidolph MR Hei-Standard, Heidolph, Schwabach, Germany,). The mixtures (100–500 mg) were placed on an aluminium plate, mixed and melted at 170 °C for one minute, after which the melt was cooled on an ice bath.

Larger-scale preparation was performed by a solvent evaporation approach. Spray drying was conducted with a Buchi Mini Spray Dryer B-191 (Buchi Labortechik AG, Flawil, Switzerland). The PMs were dissolved in MilliQ water with overnight stirring at 50 °C (500 mg in 20 mL for the 1:1 mixture or in 10 mL for the 1:2 mixture). The spray drying was performed with an outlet temperature of 160 °C. The other settings can be found in Table S1 (in Supplementary Materials). After the preparation, the co-amorphous mixtures were stored at 4 °C and 0% relative humidity (RH), which was created with P_2_O_5_ (Fisher Scientific U.K. Loughborough, UK). Co-amorphous FUR–VER and FUR–PIP were prepared by dissolving 850 mg of the PM in 50 mL of MeOH on a Petri dish. The solvent was evaporated on a hot plate (Heidolph MR Hei-Standard, Heidolph, Schwabach, Germany) at 120 °C for eight minutes. The remaining solid was collected and stored in a refrigerator at 0% RH.

### 2.2. Characterization of the Amorphous Materials

#### 2.2.1. X-ray Powder Diffraction (XRPD)

XRPD measurements were performed with a Bruker D8 DISCOVER system (Bruker AXS GmbH, Karlsruhe, Germany) using Cu Kα radiation with λ = 1.5418 Å and a motorized slit. The samples were analysed at 40 kV and 40 mA from 5 to 35° 2 θ using a scanning speed of 0.120 s/step and a step size of 0.013°. The scattered radiation was collected with a 1D LYNXEYE detector fully open.

#### 2.2.2. Differential Scanning Calorimetry (DSC)

The glass transition (T_g_, midpoint), possible recrystallization (T_rc_, onset) and melting (T_m_, onset) temperatures were measured in duplicate DSC thermograms with a TA DSC 2500 as described in Section 2.1. All the mixtures were measured between 0 and 230 °C using a 1 °C/min heating rate and temperature modulation (amplitude, ±2 °C, and frequency, 60 s). The crystalline starting materials were heated at 10 °C/min above the melting temperature (FUR: 215 °C, VER: 142 °C, and PIP: 135 °C), where they were held for five minutes, and subsequently cooled to −50 °C. After this, the samples were heated again at 10 °C/min to the temperatures mentioned above. For the ARG mixtures, DSC analysis was conducted using a Mettler Toledo DSC1 (Mettler Toledo, Schwerzenbach, Switzerland) equipped with an autosampler and a Huber TC45MT intracooler (Peter Huber Kältemaschinenbau AG, Offenburg, Germany). A nitrogen flow of 50 mL/min was used during the measurements. Temperature and heat flow calibrations were carried out with indium, lead, zinc and highly purified water standards. The measurements were performed in duplicate in 40 µL aluminium pans (Mettler Toledo, Schwerzenbach, Switzerland) sealed with a pierced lid. The amorphous samples were heated from 0 to 210 °C at 1 °C/min with temperature modulation (amplitude, ±1 °C, and frequency, 1 min). Data were collected using the STARe software (Mettler Toledo, Switzerland).

The Gordon–Taylor equation was used to calculate the theoretical T_g_ s for the mixtures (Equation (1)).

(1) $$T_{g,\mathrm{mix}}=\;\frac{W_{1}T_{g1}+{\mathrm{KW}}_{2}T_{g2}}{W_{1}+{\mathrm{KW}}_{2}}$$

where W_1_ and W_2_ are the mass fractions of the components and T_g1_ and T_g2_ are the glass transition temperatures (K) of the components. The constant K can be obtained with Equation (2):

(2) $$K\;\approx \;\frac{\rho_{1}T_{g1}}{\rho_{2}T_{g2}}$$

where ρ_1_ and ρ_2_ are the densities of the components (g/cm^3^). The densities were ρFUR = 1.61 g/cm^3^ [21], ρARG = 1.42 g/cm^3^ [15], ρVER = 1.058 g/cm^3^ [23] and ρPIP = 1.25 g/cm^3^ [24].

#### 2.2.3. Fourier-Transform Infrared Spectroscopy (FTIR)

The FTIR spectra were obtained using a Thermo Nicolet iS50 FTIR spectrophotometer (Thermo Scientific Nicolet, Madison, WI, USA) equipped with an attenuated total reflectance (ATR) accessory. Spectra were collected over the range 650–4000 cm^−1^ using a resolution of 4 cm^−1^ and taking an average of 64 scans per sample. The OMNIC 9 software (Thermo Fisher Scientific Inc. Nicolet, Madison, WI, USA) was used to collect the data.

#### 2.2.4. Stability Studies

The amorphous samples were stored under the following conditions: 4 °C/0% RH, ambient temperature (approximately 22 °C)/60% RH and 40 °C/0% RH. An RH of 60% was obtained with a saturated NaBr solution, and RH of 0%, with P_2_O_5_. The samples were analysed regularly with XRPD and FTIR, as described above.

### 2.3. Equilibrium Solubility Testing

The equilibrium solubility for FUR was measured in pH 5.2 buffer. A buffer solution of pH 5.2 was prepared according to [25] using NaOH (Fisher Scientific UK, Loughborough, UK), NaCl (Fisher Scientific) and glacial acetic acid (Riedel de Haën, Seelze, Germany). The powders (FUR or FUR PM) and 2 mL of buffer were added into the test tubes, which were placed in a shaking water bath (Grant OLS 200, Grant Instruments, Royston, UK) at 37 °C and 100 oscillations/min for three days. The pH was monitored and adjusted to 5.2 with 1 M NaOH or HCl, and powder was added if necessary. The tests were performed in triplicate for each drug and PM. The final solution was filtered through a 45 µm syringe-driven sterile filter (Syringe Filter 30 mm Dia, PES 45 μm Membrane, Sterile, Porvair Sciences), and the drug concentrations were analysed with high-performance liquid chromatography (HPLC) as described below.

### 2.4. Simultaneous Dissolution/Permeation Testing Using PAMPA

The dissolution and permeation of FUR were tested simultaneously in side-by-side diffusion cells (PermeGear Inc., Hellertown, PA, USA, with a volume of 3 mL and an effective permeation area of 0.64 cm^2^) using PAMPA membranes, according to the protocol previously described in [14,15]. Durapore PVDF (polyvinylidene fluoride) 0.1 µm membrane filters (Millipore Corporation, Bedford, MA, USA) and 15 µL of a 10% (m/V) solution of L-α-phosphatidylcholine in dodecane (both from Sigma-Aldrich, St. Louis, MO, USA) were used for the preparation of PAMPA. USP (United States Pharmacopeia) phosphate buffer, pH 5.2 (37 °C), was used as the test medium.

Powder samples corresponding to 10 mg of FUR (except for 25 mg of FUR with ARG) were placed in the donor cells, from which dissolution samples (300 µL) were withdrawn at 15, 30, 45, 60, 75, 90, 120, 150, 180, 210, 240, 270, 300, 330 and 360 min and filtered immediately through a 30 mm polyethersulfone (PES) 0.22 µm membrane filter (Guangzhou JET Bio-Filtration Co., Ltd., Guangzhou, China). Simultaneously, the acceptor cell was emptied (and discarded). The volumes removed from the cells were replaced with the buffer. The permeation test was conducted similarly but only taking samples from the acceptor cells. All the experiments were run in triplicate. The samples were diluted with acetonitrile (ACN) (3:7 ratio of sample:ACN, *v*/*v*), and the dissolution samples were diluted further with an ACN/H_2_O 70/30 solution (1:4 *v*/*v*) and analysed with HPLC as described below.

### 2.5. High-Performance Liquid Chromatography (HPLC)

Gilson HPLC equipment consisting of a Gilson 321 pump, a Gilson UV–vis 151 detector (both from Gilson Inc., Middleton, WI, USA), a Gilson 234 autoinjector (Gilson, Roissy-en-France, France) and a reversed-phase column (Phenomenex Gemini NX 5µ C18 110 A, 250 × 4.60 mm, Torrance, CA, USA) with a Phenomenex precolumn was used. The mobile phase was acetonitrile (ACN, VWR Chemicals, Fontenay-sous-Bois, France) (70%), H_2_O (Milli-Q^®^ water purification system (Merck Millipore, Darmstadt, Germany)) (30%) and trifluoroacetic acid (TFA, Sigma-Aldrich, St. Louis, MO, USA) (0.1%), with a flow rate of 1.2 mL/min. FUR was detected at a wavelength of 234 nm, and its retention time was 2.6 ± 0.02 min. A FUR standard solution of 100 µg/mL was prepared in ACN/H_2_O (70/30), from which dilutions of 0.1, 0.5, 5, 10, 25 and 50 µg/mL were prepared in ACN/H_2_O. The standard curve was linear (r^2^ = 0.9997), and the repeatability of the method (RSD) was 2.6% (25 µg/mL) and 7.6 (1 µg/mL). The results were analysed using the Gilson Unipoint software (version 3.01, Gilson Inc., Middleton, WI, USA).

### 2.6. Data Evaluation and Statistical Analysis

The area under curve (AUC) values for dissolution and permeation were calculated from the dissolution and permeation curves (between 0 and 360 min) by linear trapezoidal integration using Origin Pro 2015 64-bit (OriginLab Corporation, Northampton, MA, USA). Differences were considered significant with *p*-values < 0.05 (95% confidence level) in *t*-tests (ANOVA single-factor).

## 3. Results and Discussion

### 3.1. Selection of Co-Formers and Preparation Method for Co-Amorphous Formulations

Piperine (PIP, a plant alkaloid), verapamil hydrochloride (VER, an antihypertensive drug compound) and quercetin (QRT, a plant-derived flavonoid) were selected as co-formers for FUR, as they can inhibit P-gp-mediated efflux and thus potentially improve a drug’s bioavailabilty when co-administered orally with P-gp substrates [1,22,26]. ARG was selected based on previous research showing the ability for co-amorphization and salt formation with FUR [27,28]. However, when selecting the preparation methods for amorphous formulations of FUR, it should be considered that FUR experiences thermal decomposition upon melting [17], it is poorly water soluble [18], and it is easily degradable upon milling [19,20]. We attempted the cryo-milling of FUR alone and with the co-formers of ARG, PIP, QRT and VER. In all of the cases, milling resulted in the easily observable (colour change and smell) degradation of FUR, raising doubts about the degree of degradation upon the co-amorphization of FUR by milling with amino acids in previous studies [21,28]. The milled products were not analysed further. Instead, the co-melting of FUR with the co-formers was attempted, since it is known that miscible compounds exhibit lowered common melting points, and that this would potentially be one way of preventing the thermal decomposition of FUR. It was observed by DSC that FUR was miscible with VER and PIP with 1:1 and 1:2 molar ratios, showing common and lowered melting temperatures (the results are shown in the Supplementary Materials, Figure S1). It has been reported that FUR melts and decomposes at 217–222 °C [29]. With VER, the melting point of the mixture was lowered to approx. 127 °C (with both molar ratios), and with PIP, to approx. 111 °C (with both molar ratios), while the melting points of pure VER and PIP were 139.9 ± 0.64 and 130.1 ± 0.04 °C, respectively. Thus, we attempted the co-melting and rapid cooling of the formulations, but again, evidence of decomposition (the appearance of a brown colour) was observable with the naked eye immediately after melting. Thus, these methods were excluded from further consideration. Spray drying from aqueous ARG solutions and the evaporation of methanol solutions of FUR with VER, PIP and QRT were selected as the preparation methods.

### 3.2. Solid-State Characterization of the Co-Amorphous Formulations

#### 3.2.1. X-ray Powder Diffraction

**Figure 1:**
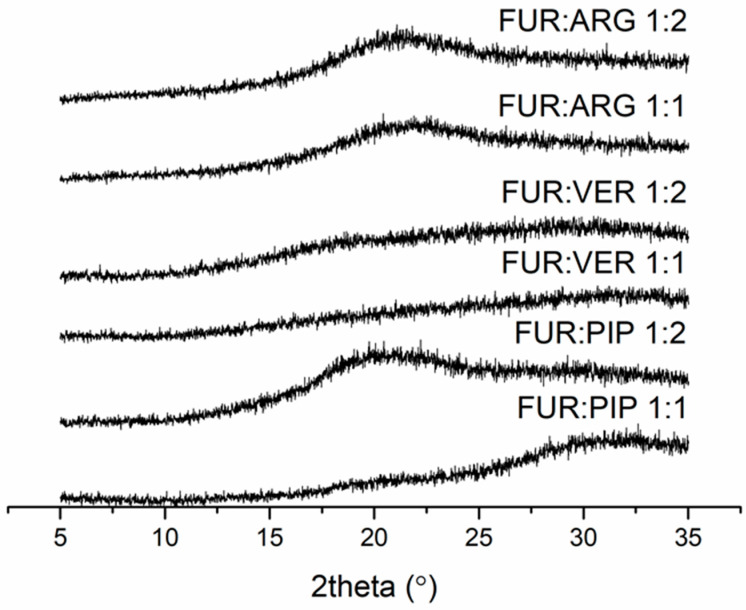
X-ray diffractograms of furosemide (FUR):arginine (ARG) and FUR:D-ARG at 1:1 and 1:2 molar ratios prepared by spray drying, and FUR:piperine (PIP) and FUR:verapamil HCl (VER) mixtures at 1:1 and 1:2 molar ratios prepared by the evaporation method.

The FUR mixtures with ARG (prepared by spray drying), PIP and VER (prepared by evaporation) at 1:1 and 1:2 molar ratios were amorphous according to XRPD (Figure 1). Instead, the mixtures with QRT (prepared by evaporation) remained crystalline at both molar ratios (the X-ray diffractograms along with those of the starting materials are shown in Figure S2 in the Supplementary Materials).

#### 3.2.2. Differential Scanning Calorimetry

The experimental T_g_ values, theoretical T_g_ values obtained with the Gordon–Taylor equation, and possible recrystallization (T_rc_) and melting (T_m_) temperatures observed for the materials are shown in Table 1. Example thermograms are shown in the Supplementary Materials (Figures S3 and S4). It was observed that all the co-amorphous mixtures displayed single T_g_ values that were higher than expected based on the Gordon–Taylor equation, except for FUR:VER 1:2. This indicates that the mixtures were homogenous single-phase systems and, furthermore, that intermolecular interactions may have been formed between the components [30,31]. Instead, the FUR:VER 1:2 most probably consisted of a VER-rich phase with a lower T_g_ value and a FUR-rich phase with a higher T_g_. The co-amorphous mixtures with ARG as co-formers displayed considerably higher T_g_ values than theoretically predicted, i.e., indicative of salt formation between the components (1:1 molar ratio), as expected [21,28]. The T_g_ of the FUR:ARG system has previously been observed to have the highest value at a 1:1 molar ratio due to salt formation [21]. The values for the spray-dried FUR:ARG mixtures were also in accordance with those for the FUR:ARG mixtures prepared by milling [21,28], despite the possibility for FUR decomposition during milling [19,20]. The FUR:ARG 1:2 system showed a significantly lower T_g_ than the 1:1 system (Table 1), which indicates that at the 1:2 molar ratio, ARG is present as an excess amorphous component (miscible with the salt), and no further interactions occur between the co-amorphous salt and the excess ARG [21].

#### 3.2.3. Fourier-Transform Infrared Spectroscopy

**Figure 2:**
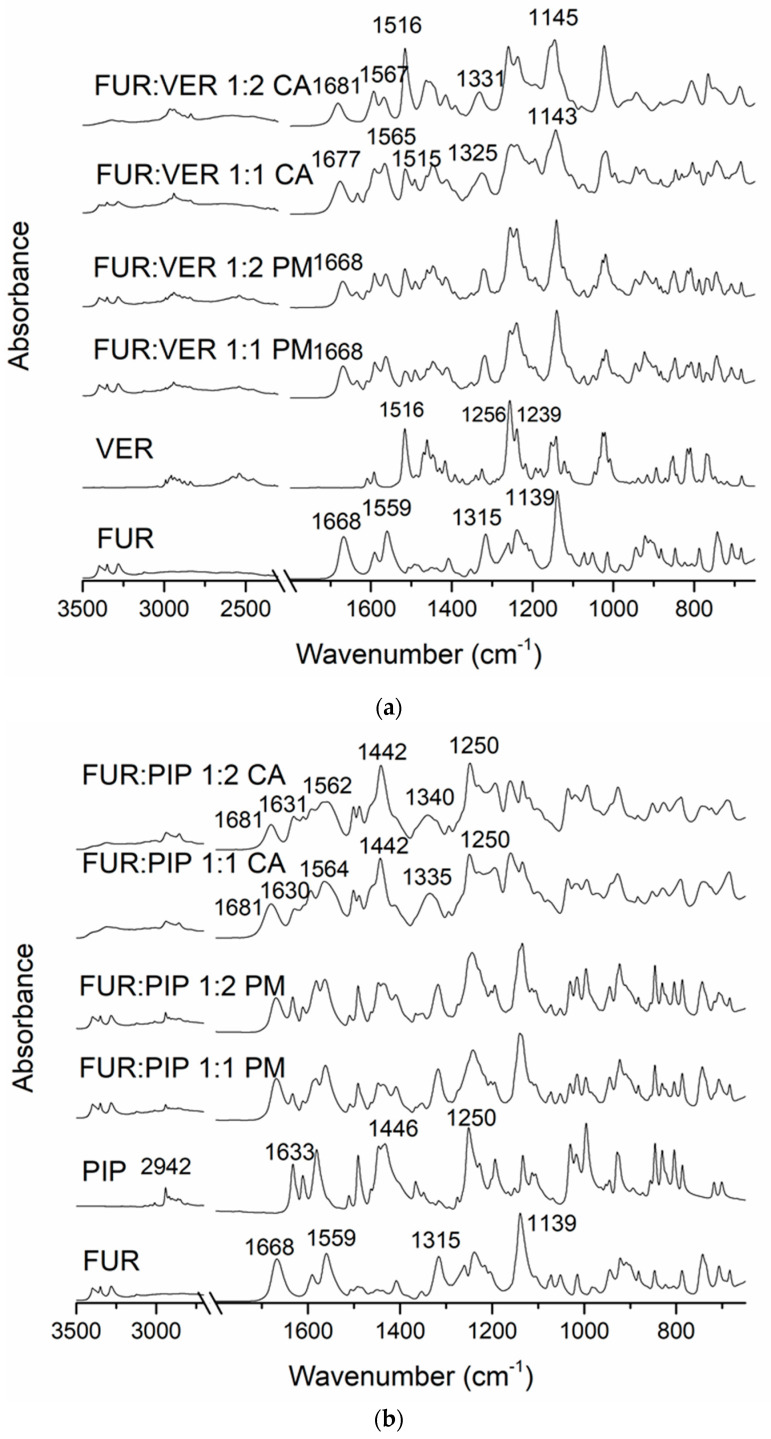

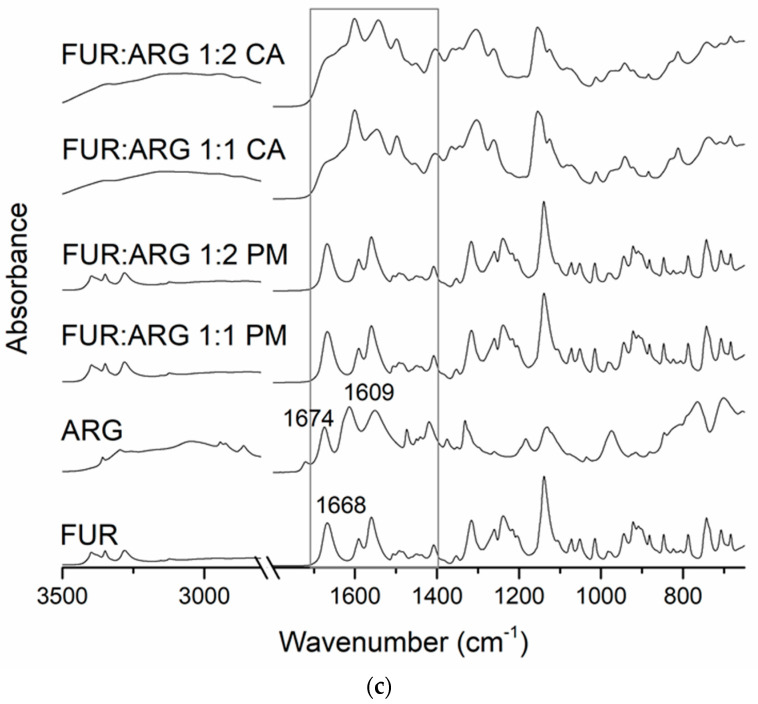
The Fourier-transform infrared (FTIR) spectra of (**a**) crystalline furosemide (FUR) and verapamil HCl (VER), their physical mixtures (PMs) at 1:1 and 1:2 molar ratios and the corresponding co-amorphous (CA) mixtures; (**b**) crystalline FUR and piperine (PIP), their PMs at 1:1 and 1:2 molar ratios and the corresponding CA mixtures; (**c**) crystalline FUR and arginine (ARG), their PMs and the corresponding CA mixtures at 1:1 and 1:2 molar ratios, with a rectangle showing the area for spectral changes due to salt formation.

In the FTIR spectra of the crystalline FUR and the physical mixtures with VER (Figure 2a), the NH_2_ stretching of the Ar-NHCH_2_-group at 3349 cm^−1^, the NH_2_ stretching of the SO_2_NH_2_ group at 3280 cm^−1^, the stretching vibration of SO_2_NH_2_ at 1668 cm^−1^ and the asymmetric stretching vibration of the carboxyl group at 1559 cm^−1^ can be seen [34]. The carboxyl peak shift does not occur in pure amorphous FUR [32]; thus, this may indicate an interaction with VER in the co-amorphous mixtures. No peak shifts were observed in the physical mixtures. In the co-amorphous FUR:VER 1:1 and 1:2 mixtures (Figure 2a), these peaks had shifted to higher wavenumbers, i.e., to 1677 and 1681 cm^−1^, and 1565 and 1567 cm^−1^, respectively. The peaks at 1315 cm^−1^ (asymmetric SO_2_ stretching) and 1139 cm^−1^ (symmetric SO_2_ stretching) in FUR [35] had shifted to 1325 and 1331 cm^−1^, and to 1143 and 1145 cm^−1^ in the co-amorphous FUR:VER 1:1 and 1:2, respectively.

The spectrum of VER shows the characteristic absorptions at approx. 3000–2800 cm^−1^ (C-H of the methoxy groups); 2700–2400 cm^−1^ (N-H of protonated amine); 1608, 1591 and 1516 cm^−1^ (aromatic C=C); and 1256 and 1239 cm^−1^ (C-O-C of aromatic esters) [36]. The disappearance of the amine peaks in the co-amorphous mixtures with FUR may point to a hydrogen-bonding interaction with FUR.

The spectrum of PIP (Figure 2b) shows the characteristic peaks at 2942 cm^−1^ (aromatic C-H stretching), 2854 cm^−1^ (C-H stretching of methylenedioxy group), 1633 cm^−1^ (C=0 stretching), 1610 cm^−1^ (symmetric stretching of C=C of aliphatic diene), 1446 cm^−1^ (aromatic C=C stretching) and 1250 cm^−1^ (asymmetric =C-O-C stretching) [37]. When co-amorphous mixtures with FUR were formed, we observed the disappearance of the FUR amine peaks at approx. 2300 cm^−1^. In addition, the FUR peak (amino) at 1668 cm^−1^ shifted to 1681 cm^−1^, which is an even larger shift than that observed with the co-amorphous mixtures with VER. The sulphonyl peak of FUR at 1315 cm^−1^ shifted to 1335 cm^−1^ (1:1) and 1340 cm^−1^ (1:2) in the co-amorphous mixtures. The changes in PIP absorptions were more modest, but the aromatic C=C stretching peak at 1446 cm^−1^ shifted to 1442 cm^−1^ in both co-amorphous mixtures, and the aromatic C-H stretching at 2942 cm^−1^ broadened and reduced in intensity. These changes may indicate intermolecular interactions between the components.

In the case of the ARG mixtures (Figure 2c), salt formation was observed, as expected. The disappearance of the unbound COOH group signal of FUR (at 1668 cm^−1^ in crystalline FUR) together with changes in the guanidine structure of ARG (1674 and 1609 cm^−1^ in crystalline ARG) have previously been reported as confirmation of salt formation between the components [21,28].

#### 3.2.4. Physical Stability

**Figure 3:**
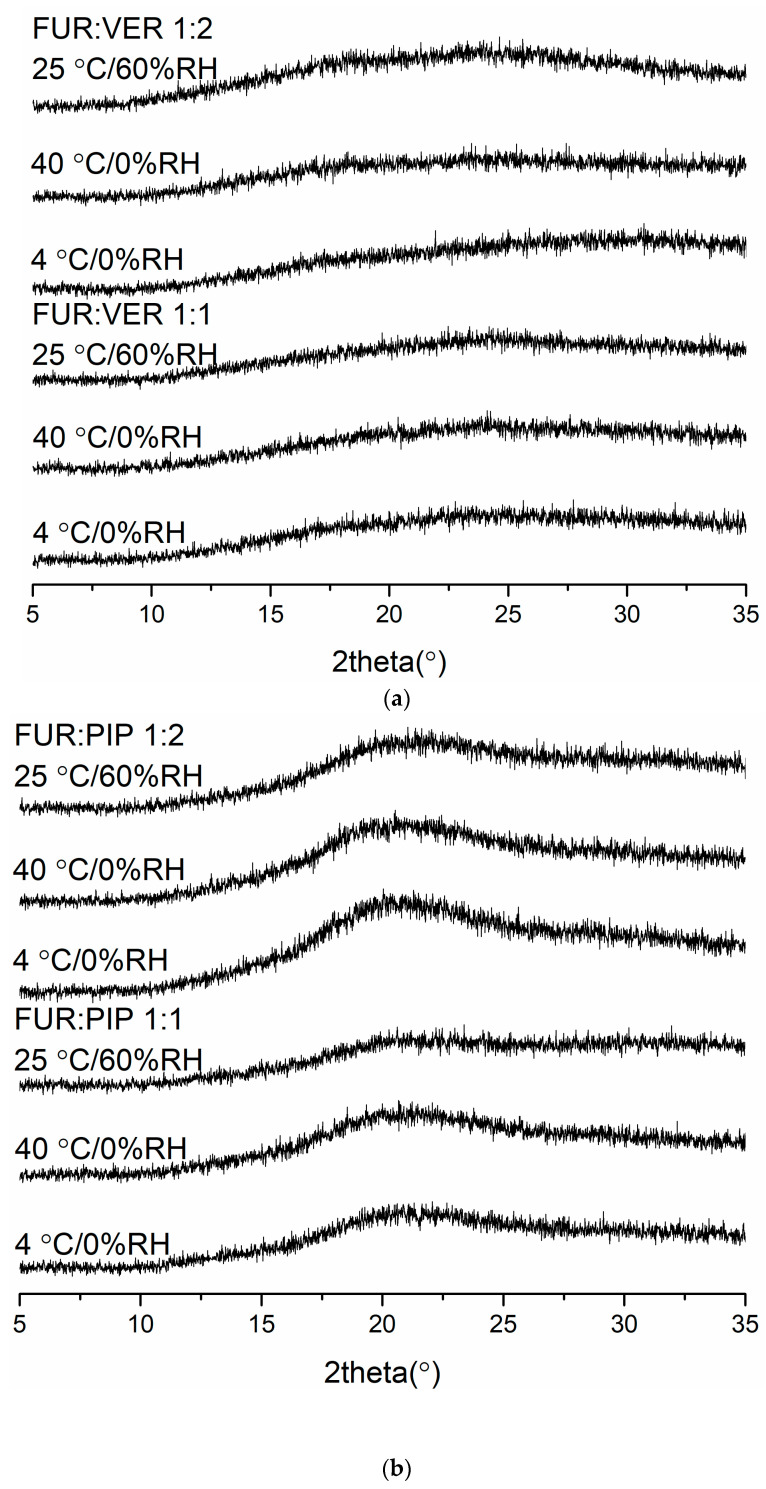

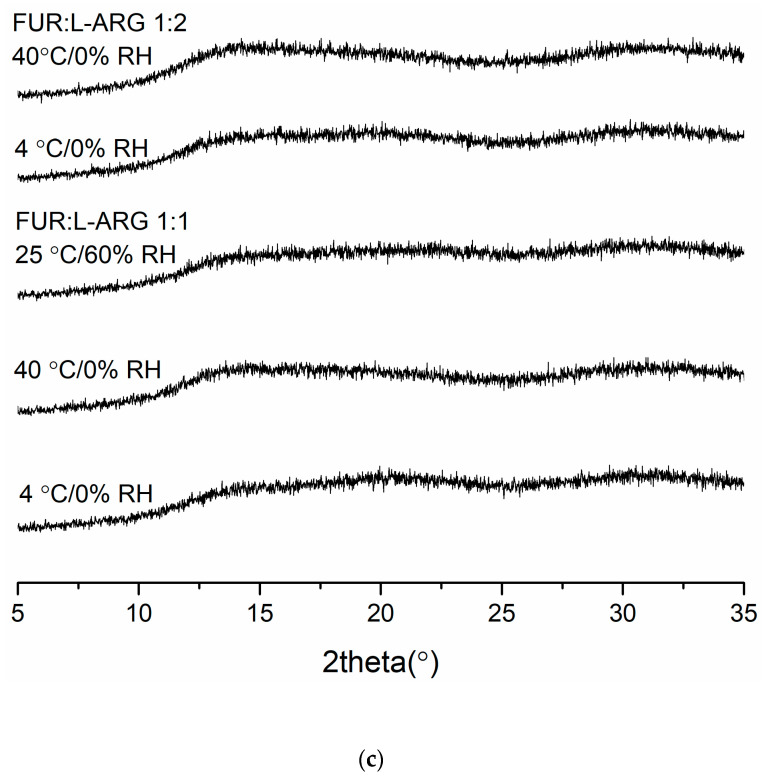
The X-ray diffractograms of (**a**) furosemide:verapamil HCl (FUR:VER) co-amorphous mixTable 1. and 1:2 molar ratios stored at 4 °C/0% RH, 40 °C/0% RH and 25 °C/0% RH for 2 months; (**b**) furosemide:piperine (FUR:PIP) co-amorphous mixtures at 1:1 and 1:2 molar ratios stored at 4 °C/0% RH, 40 °C/0% RH and 25 °C/0% RH for 2 months; and (**c**) furosemide:arginine (FUR:ARG) co-amorphous mixtures at 1:1 and 1:2 molar ratios stored at 4 °C/0%RH, 40 °C/0% RH and 25 °C/0% RH for 9 weeks.

The X-ray diffractograms of the co-amorphous mixtures stored at 4 °C/0% RH, 40 °C/0% RH and 25 °C/60% RH are shown in Figure 3. The samples were found to be physically stable during the entire storage period (eight weeks for FUR:VER and FUR:PIP, and nine weeks for FUR:ARG). However, when the FUR:ARG 1:2 mixture was stored at 25 °C/60% RH, it transformed into a sticky material in less than one week; thus, it was not investigated further. Similar hygroscopic behaviour has been observed with other amorphous ARG salts, such as spray-dried ibuprofen:ARG and indomethacin ARG [38]. Furthermore, the FTIR spectra (Figure S5) also support the XRPD results, as none of the mixtures exhibited any changes during storage. Thus, all the mixtures were able to stabilize amorphous FUR, as the pure drug has been found to recrystallize within two weeks when stored at 25 or 40 °C [28] and four days when stored at 22 °C/33% RH [39]. This could be attributable to the increased T_g_ values of the samples and intermolecular interactions between the components [2,40], especially salt formation between the ARGs and FUR [28,38].

### 3.3. Solubility and Simultaneous Dissolution/Permeation

The equilibrium solubility of FUR was found to be 0.504 ± 0.05 mg/mL at a pH of 5.2 (Table 2), and it increased almost 100 fold when the pH was raised to 7.4 (results not shown). Thus, pH 5.2 was used in the solubility and dissolution tests. It was found that PIP and VER had a negative effect on FUR solubility (except for FUR:PIP 1:2, Table 2). Instead, ARG slightly increased FUR solubility, which has been reported to be due to salt formation [21,28,41].

**Figure 4:**
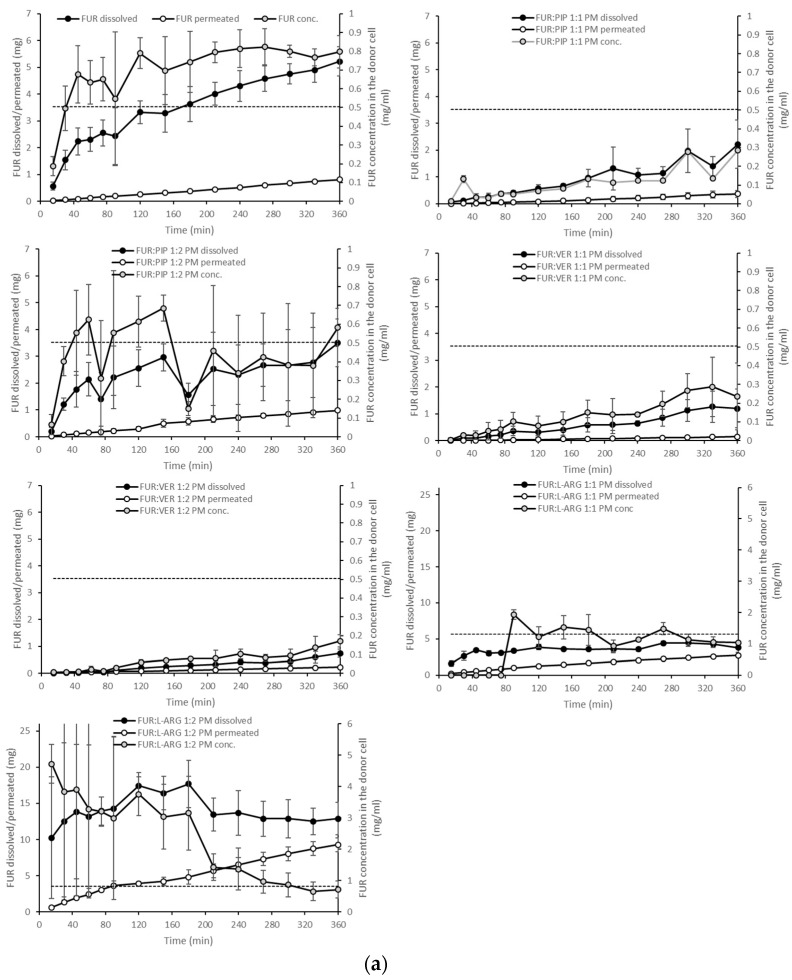

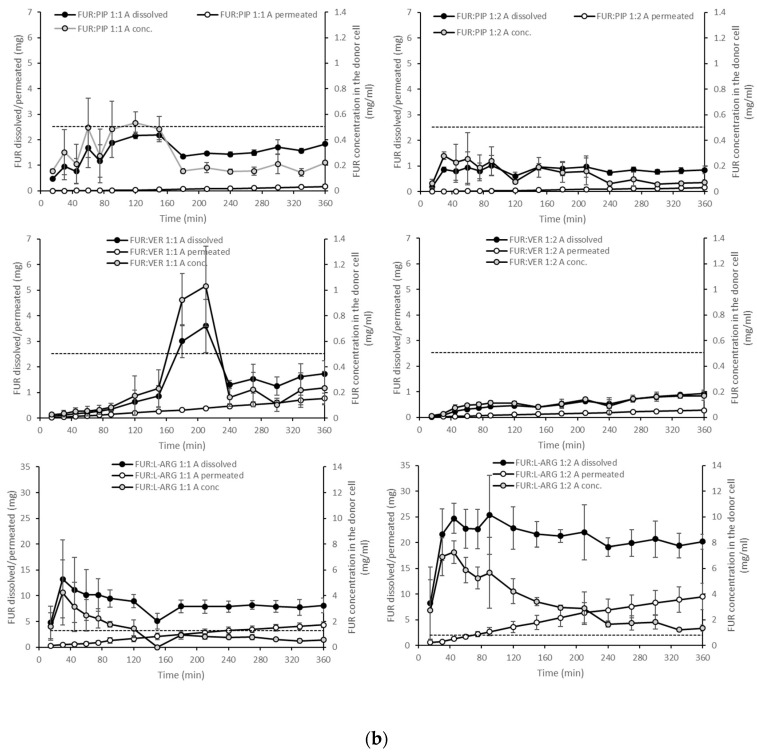
Cumulative amounts of furosemide dissolved and permeated (mg, *n* = 3 ± sd.) in the simultaneous dissolution/permeation test (left axis). Furosemide concentrations in the donor cell (mg/mL) and the equilibrium solubility of furosemide (right axis): (**a**) crystalline furosemide (FUR) and physical mixtures with piperine (FUR:PIP), verapamil hydrochloride (FUR:VER) and l-arginine (FUR:ARG) at 1:1 and 1:2 molar ratios, and (**b**) amorphous FUR:PIP, FUR:VER and FUR:ARG mixtures at 1:1 and 1:2 molar ratios.

The dissolution test showed clearly that in the case of the PMs, the dissolution of FUR from the FUR:PIP and FUR:VER mixtures was lower when compared to that with crystalline FUR alone (Figure 4a). This is in accordance with the results from the equilibrium solubility tests (Table 2). Instead, ARG had a positive effect on the dissolved amounts of FUR. It seems that the equilibrium solubility was exceeded in the FUR:ARG 1:2 mixture. After reaching supersaturation, the FUR concentrations declined back to the level of equilibrium solubility. This may be due to in situ amorphization, which has been observed to occur for ARG and different drug molecules [27,42].

With the amorphous mixtures, the behaviour of the FUR:PIP and FUR:VER mixtures was similar to that of the corresponding PMs, and supersaturation was not reached (Figure 4b). This may be due to the powder properties; i.e., the FUR:PIP and FUR:VER mixtures prepared by solvent (MeOH) evaporation became sticky upon contact with the dissolution medium, causing the powder to adhere to the magnetic stirrer, which even prevented the stirrer from moving in some cases. This behaviour may have negated the possible dissolution advantage created by the amorphous state. In addition, FUR:VER 1:1 showed a sudden and unexplained increase in the dissolved amount of FUR being observed between 150 and 240 min. Previously, PIP has been reported to enhance the dissolution of poorly soluble curcumin; i.e., a co-amorphous curcumin:PIP mixture was able to provide over threefold supersaturation at pH 6.8 when compared to crystalline curcumin [24].

Amorphization provided a further increase in FUR dissolution for the amorphous mixtures with ARG (Figure 4b). More specifically, FUR:ARG 1:2 showed the highest amounts of dissolved FUR, i.e., 25 mg (at 45 min), which represented approx. nine-fold supersaturation. This supersaturation was also long-lasting, as it prevailed for the whole duration of the test (6 h). The FUR:ARG 1:1 mixture showed a more modest supersaturation, i.e., approx. three-fold (at 30 min). This is the typical behaviour of co-amorphous ARG salts, as observed, for example, with hydrochlorothiazide [15]. As the co-amorphous mixtures with ARG were prepared by a different method (spray drying) than the mixtures with VER and PIP (solvent evaporation), differences in powder properties, such as the particle size and surface area, may have had their own impacts on the powder dissolution rate.

When examining the permeating amounts, the lowest total permeated amounts of FUR from crystalline materials were observed for FUR:VER 1:1 (0.15 mg), which correlates well with the dissolved amounts and the concentration gradient driving the passive permeation (Figure 4a). With the PMs containing ARG, the total permeating amounts were significantly higher when compared to crystalline FUR (0.81 mg), i.e., 2.7 and 9.3 mg for FUR:ARG 1:1 and FUR:ARG 1:2, respectively. In the case of the co-amorphous mixtures, the total permeating amounts of FUR for the PIP and VER mixtures were similar or even lower than with the corresponding PMs. Once again, the mixtures with ARG provided the highest total permeating amounts, i.e., 4.3 and 9.5 for FUR:ARG 1:1 and FUR:ARG 1:2, respectively.

The AUC values for the dissolution and permeation of the different mixtures are shown in Table 3. The table also displays a comparison of the AUC values of the mixtures against those of crystalline FUR and the AUC values of the co-amorphous mixtures against those of the PMs. From the table, it is evident that only ARG (at a molar mixture of 1:2) was able to significantly enhance the dissolution of FUR in PMs, when compared to the dissolution of crystalline FUR. The PMs containing PIP and VER showed significantly smaller AUC values than crystalline FUR, which is in accordance with the equilibrium solubility results (Table 2). The same comparison conducted for permeation reveals that permeation was enhanced with the FUR:PIP 1:2 PM and FUR:ARG PMs. With ARG, the enhanced permeation is only partly explainable by the increased concentration gradient. When comparing the AUC values of the co-amorphous mixtures against those of crystalline FUR, only the FUR:ARG mixtures displayed significantly increased AUC values. The same can be observed with permeation, but the increase in AUC values was, again, larger than that for dissolution, indicating that different permeation-enhancing factors other than the increased concentration gradient may be involved. Finally, when comparing the co-amorphous mixtures against the corresponding PMs, it can be observed that amorphization significantly enhanced dissolution with the FUR:VER and FUR:ARG mixtures (both 1:1 and 1:2). Permeation was significantly enhanced for FUR:VER 1:1 and FUR:ARG 1:1 and 1:2 with respect to their corresponding PMs.

**Figure 5:**
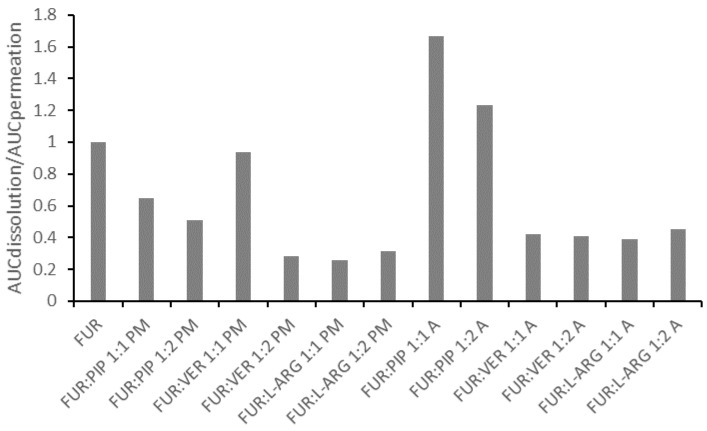
The ratio between the areas under curves for dissolution (AUCdissolution) and for permeation (AUCpermeation) for the mixtures, divided by the ratios, for crystalline FUR.

In general, the correlation between the AUC values for dissolution and permeation was linear (Figure S7 in the Supplementary Materials); thus, the increased AUC for dissolution led to an increase in permeation. This observation is in line with findings previously reported for formulations of glibenclamide and hydrochlorothiazide in similar PAMPA experiments [14,15]. However, when the ratio of the AUC for the dissolution to the AUC for the permeation of the mixtures is normalized against that for crystalline FUR, it is evident that all those formulations having ratios smaller than one are the PMs and the co-amorphous mixtures with VER and ARG (Figure 5). This means that for these formulations, permeation occurred more efficiently in relation to dissolution, when compared to the case for crystalline FUR. As stated previously, the results for the FUR:ARG PMs and co-amorphous mixtures indicate that some permeation-enhancing factors other than an increased concentration gradient may be involved. It has been previously postulated that ARG may make the PAMPA membrane more permeable by undertaking interactions with lecithin (stacking interactions between the guanidium moieties) [14,43]. For the co-amorphous FUR:PIP mixtures; the ratio was greater than one, indicating that passive permeation was less efficient in relation to dissolution when compared to the case for crystalline FUR.

As PIP and VER are known P-gp inhibitors, it would be essential to also study permeation in relevant cell models in order to reveal their possible permeation-enhancing effects [16,44,45]. However, with FUR being relatively well soluble at a neutral pH, a condition that is preferred in cellular membrane permeation experiments [46], it may be difficult to conduct simultaneous dissolution/permeation testing with cultured cells. The amount of powder mixture required for enabling supersaturation would be a hundred times higher at pH 7.4 than at pH 5.2 (according to the equilibrium solubility testing), which may be excessive considering the donor cell volume (3 mL) and, also, probably too toxic to the cells. It would be possible to test the permeation enhancement in a traditional way using supersaturated (e.g., via solvent shift) solutions of FUR and excipients, but then, the effects of the solid-state properties of a formulation (amorphization and powder properties) and the different dissolved states on the dynamic permeation process would remain unrevealed [45,47,48]. To provide further insights into the absorption potential of the FUR mixtures, in vivo testing may be the preferred next step.

## 4. Conclusions

Co-amorphous mixtures of FUR were prepared with the amino acid ARG (by spray drying) and P-gp inhibitors VER and PIP (by solvent evaporation) at 1:1 and 1:2 molar ratios. All the co-amorphous mixtures showed single T_g_s that were higher than the values predicted by the Gordon–Taylor equation, except for FUR:VER 1:2, indicative of the formation of homogenous single-phase systems and intermolecular interactions between the components. Instead, FUR:VER 1:2 most probably consisted of two phases (VER-rich and FUR-rich). The co-amorphous mixtures with ARG showed considerably higher T_g_ values than theoretically predicted, indicative of the presence of salt formation between the components (1:1 molar ratio). However, for FUR:ARG 1:2, the T_g_ was significantly lower than for the 1:1 mixture due to ARG being present as an excess amorphous component. Intermolecular interactions between the co-amorphous components, particularly salt formation between FUR and ARG, were verified by FTIR measurements. Increased T_g_ values and stabilizing intermolecular interactions improved the physical stability of the co-amorphous mixtures at storage conditions of 4 °C/0% RH, 40 °C/0% RH and 25 °C/60% RH for at least two months, except for FUR:ARG 1:2, which became sticky at 25 °C/60% RH.

In the simultaneous dissolution/permeation studies, the co-amorphous mixtures prepared by solvent evaporation (VER and PIP as co-formers) did not increase the dissolved amounts or AUC values for dissolution and, furthermore, did not elevate the permeating amounts of FUR or its AUC value for permeation, when compared to the case for the crystalline drug, possibly due to the poor powder properties when the powder came into contact with the dissolution medium. Instead, when a co-amorphous mixture was prepared with ARG, all the above-mentioned properties were enhanced in comparison to those of crystalline FUR. However, when examining the ratio between the dissolution and permeation of FUR, permeation occurred more efficiently in relation to dissolution with the co-amorphous mixtures with VER and ARG. This may indicate that different permeation-enhancing factors other than an increased concentration gradient may be involved, and thus, the further clarification of the dissolution/permeation interplay for these formulations would be enlightening.

## Figures and Tables

**Table 1:** Glass transition (T_g_), recrystallization (T_rc_) and melting (T_m_) temperatures of furosemide (FUR), l- and d-arginine (ARG), verapamil hydrochloride (VER) and piperine (PIP) and their co-amorphous mixtures at 1:1 and 1:2 molar ratios.

Material	T_g_ (°C) (Experimental)	T_g_ (°C) (Theoretical)	T_rc_ (°C)	T_m_ (°C)
FUR	71.1 ± 0.18	61.8 ^1^	-	-
ARG ^2^	-	55.0	-	211.1 ± 236.1
VER	56.9 ± 0.21	61.0 ^3^	118.2 ± 1.12	139.9 ± 0.64
PIP	11.4 ± 1.03	15.5 ^4^	89.4	130.1 ± 0.04
FUR:ARG 1:1	121.5 ± 0.92	64.9 ^5^	-	-
FUR:ARG 1:2	103.8 ± 0.55	62.1 ^5^	-	-
FUR:VER 1:1	68.0 ± 2.14	61.1 ^5^	-	-
FUR:VER 1:2	51.3 ± 0.95; 69.0 ± 2.64 ^6^	59.4 ^5^	-	-
FUR:PIP 1:1	48.6 ± 1.70	36.9 ^5^	-	-
FUR:PIP 1:2	42.6 ± 0.33	27.6 ^5^	-	-

^1^ Prepared by spray-drying [32]. ^2^ data from [15,21]. ^3^ prepared by melt-quenching [33]. ^4^ [24]. ^5^ evaluated with Gordon–Taylor equation. ^6^ possible second T_g_—not detected.

**Table 2:** Equilibrium solubility of FUR at pH 5.2 (*n* = 3).

Sample	Solubility (mg/mL)
FUR	0.504 ± 0.05
FUR:PIP 1:1	0.451 ± 0.12
FUR:PIP 1:2	0.612 ± 0.05
FUR:VER 1:1	0.228 ± 0.06
FUR:VER 1:2	0.035 ^1^
FUR:L-ARG 1:1	1.31 ± 0.511
FUR:L-ARG 1:2	0.825 ± 0.412

^1^ FUR was detected only in one parallel sample.

**Table 3:** Area under curve (AUC, mg * min) values for dissolved and permeated FUR during the 6 h dynamic dissolution/permeation test.

	FUR	FUR:PIP 1:1	FUR:PIP 1:2	FUR:VER 1:1	FUR:VER 1:2	FUR:L-ARG 1:1	FUR:L-ARG 1:2
Crystalline FUR or physical mixtures							
Dissolution	1250 ± 159	332 ± 100	833 ± 204	209 ± 87	104 ± 24	1265 ± 87	5062 ± 849
Permeation	142 ± 6	58 ± 25	186 ± 16	25 ± 5	41 ± 2	562 ± 16	1836 ± 210
Co-amorphous mixtures							
Dissolution	-	381 ± 175	286 ± 54	460 ± 111	186 ± 13	2889 ± 613	7320 ± 965
Permeation	-	26 ± 10	26 ± 21	124 ± 24	51 ± 8	848 ± 198	1847 ± 542
AUC ratio, physical mixtures vs. crystalline FUR							
Dissolution	-	0.27 ^2^	0.67 ^2^	0.17 ^2^	0.08 ^2^	1.01	4.05 ^1^
Permeation	-	0.41 ^2^	1.31 ^1^	0.18 ^2^	0.29 ^2^	3.96 ^1^	12.95 ^1^
AUC ratio, co-amorphous mixtures vs. crystalline FUR							
Dissolution	-	0.30 ^2^	0.23 ^2^	0.37 ^2^	0.15 ^2^	2.31 ^1^	5.85 ^1^
Permeation	-	0.18 ^2^	0.19 ^2^	0.87	0.36 ^2^	5.98 ^1^	13.03 ^1^
AUC ratio, co-amorphous vs. corresponding physical mixture							
Dissolution	-	1.15	0.34 ^2^	2.20 ^1^	1.79 ^1^	2.28 ^1^	1.45 ^1^
Permeation	-	0.45	0.14 ^2^	4.89 ^1^	1.25	1.51	1.01

^1^ Statistically significantly higher. ^2^ Statistically significantly lower.

## Data Availability

The data are contained within the article and the Supplementary Materials.

## Supplementary Materials

The following are available online at https://www.mdpi.com/1999-4923/13/2/171/s1. Figure S1: Example thermograms of furosemide:verapamil HCl (FUR:VER), furosemide:piperine (FUR:PIP), furosemide:quercetin (FUR:QRT) mixtures and furosemide:arginine (FUR:L-ARG) at 1:1 and 1:2 molar ratios. Figure S2: X-ray diffractograms of (a) the crystalline starting materials furosemide (FUR), piperine (PIP) and verapamil hydrochloride (VER); (b) crystalline quercetin (QRT) and FUR:QRT 1:1 and 1:2 molar mixtures prepared by the evaporation method. Figure S3: Example thermograms (second heating cycle) of the crystalline starting materials furosemide (FUR), verapamil hydrochloride (VER) and piperine (PIP) showing glass transitions (Tg, midpoint). Figure S4: Example thermograms of the co-amorphous mixtures of furosemide (FUR), verapamil hydrochloride (VER), piperine (PIP) and arginine (ARG) at 1:1 and 1:2 molar ratios. Figure S5: Fourier-transform infrared (FTIR) spectra of the co-amorphous materials after storage at 4 °C/0% RH, 40 °C/0% RH and 25 °C/60% RH. Figure S6: The correlation between the AUC values for dissolution and permeation. Table S1: Outlet temperatures, inlet temperatures, pump rates and flow rates for the co-amorphous mixtures prepared by spray drying.
