# Supplementary material for: Co-Amorphous Formulations of Furosemide with Arginine and P-Glycoprotein Inhibitor Drugs
Source: Pharmaceutics. 2021 Jan 27;13(2):171. doi: 10.3390/pharmaceutics13020171 (PMC7912042; doi:10.3390/pharmaceutics13020171)
Supplement: Supplementary file 1 [file pharmaceutics-13-00171-s001.pdf]

pharmaceutics

Supplementary Materials: Co-Amorphous Formulations of Furosemide with Arginine and P-Glycoprotein Inhibitor Drugs

Marika Ruponen, Konsta Kettunen, Monica Santiago Pires and Riikka Laitinen

Table S1. Outlet temperatures, inlet temperatures, pump rates and flow rates for the co-amorphous mixtures prepared by spray drying.

**Mixture**	Outlet temperature (°C)	Inlet temperature (°C) (min-max)	Pump rate (%) (min-max)	Flow rate (ml/min) (min-max)
FUR + L-ARG 1:1	160	91-94	27-30	2.94-5.87
FUR + L-ARG 1:2	160	82-86	10-30	2.13-6.17

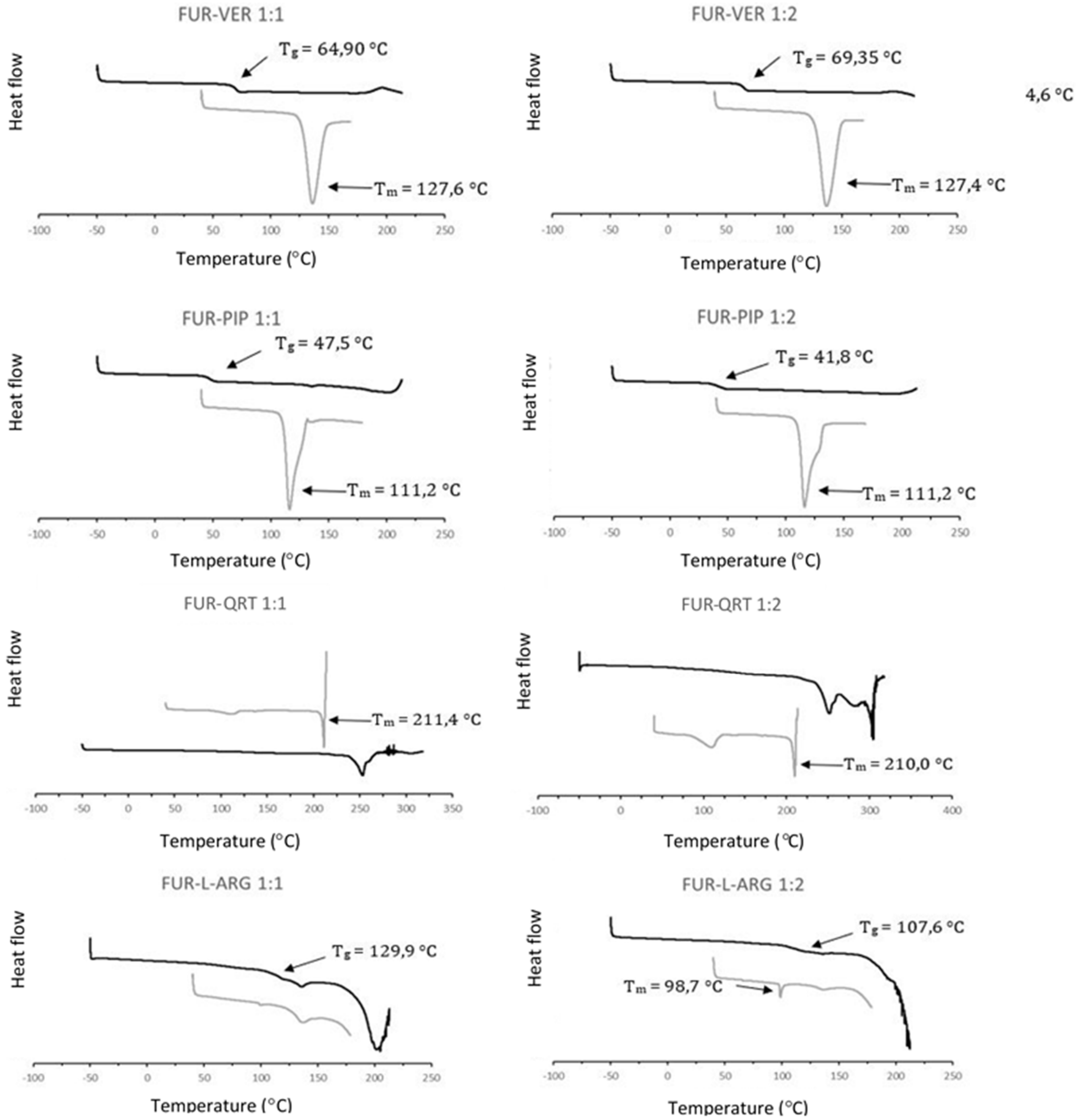

Figure S1. Example thermograms of furosemide:verapamil HCl (FUR:VER), furosemide:piperine (FUR:PIP), furosemide:quercetin (FUR:QRT) mixtures and furosemide:arginine (FUR:L-ARG) at 1:1 and 1:2 molar ratios.

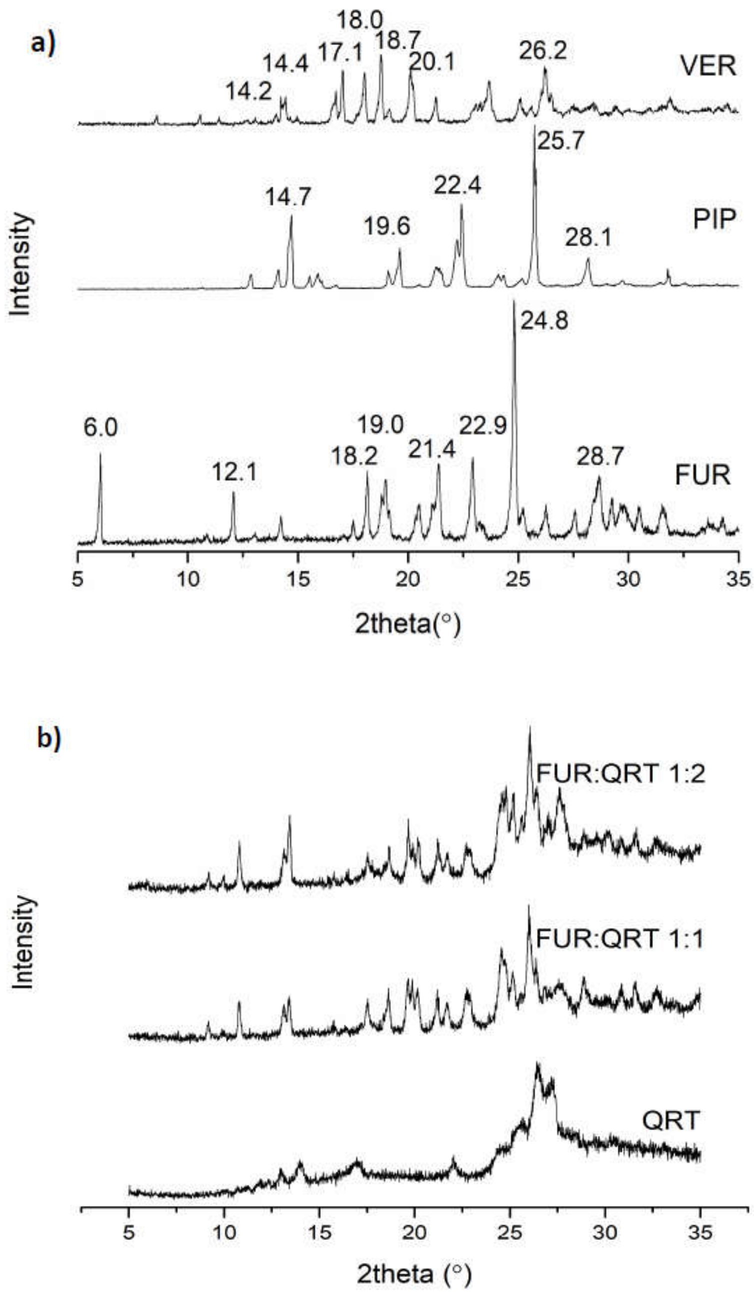

Figure S2. X-ray diffractograms of a) the crystalline starting materials furosemide (FUR), piperine. (PIP) and verapamil hydrochloride (VER). Diffractogram of ARG has been published previously by Ruponen et al., 2018 and 2020; b) crystalline quercetin (QRT) and FUR:QRT 1:1 and 1:2 molar mixtures prepared by the evaporation method.

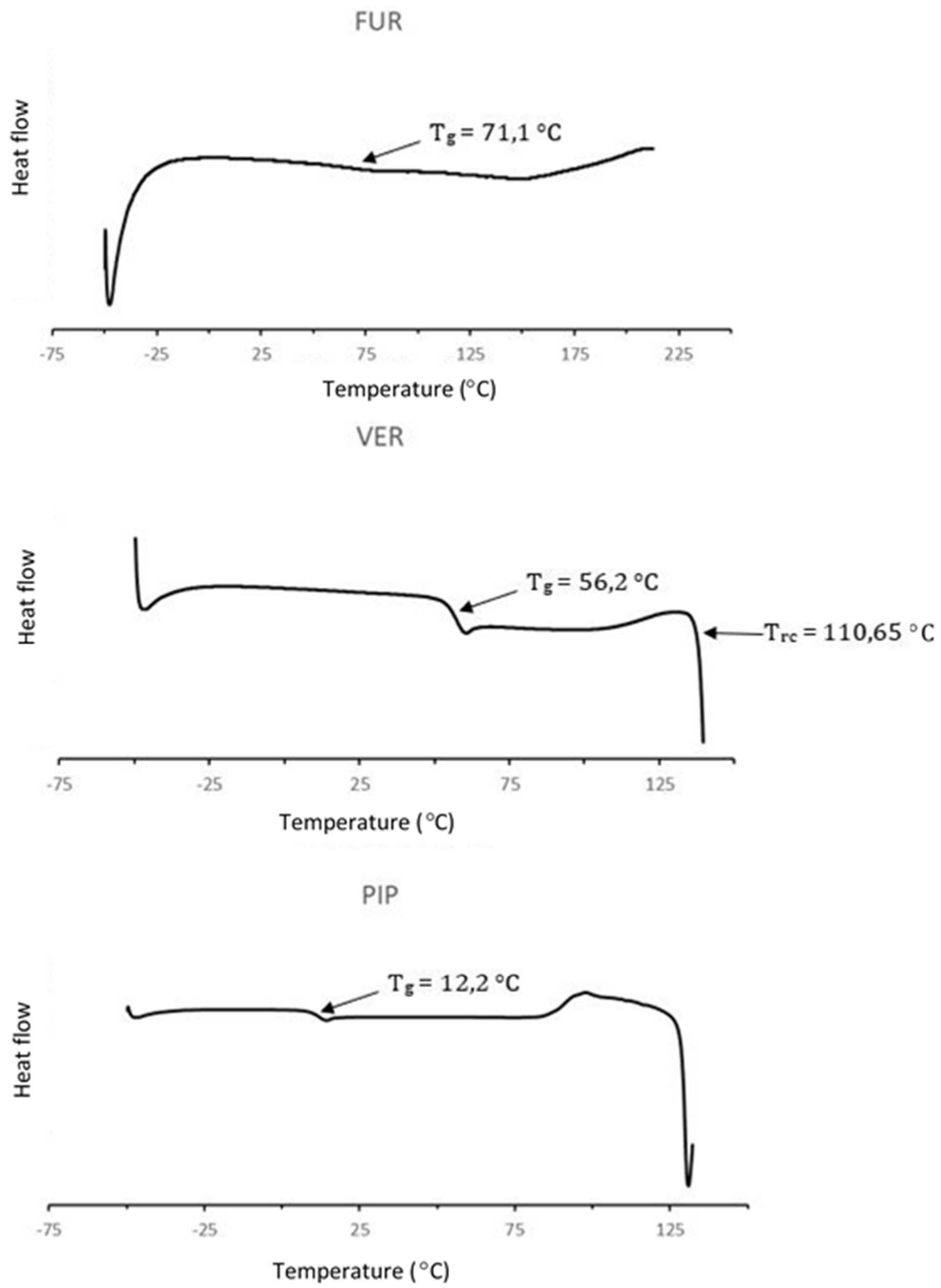

Figure S3. Example thermograms (second heating cycle) of the crystalline starting materials furosemide (FUR), verapamil hydrochloride (VER) and piperine (PIP) showing glass transitions (Tg, midpoint).

.

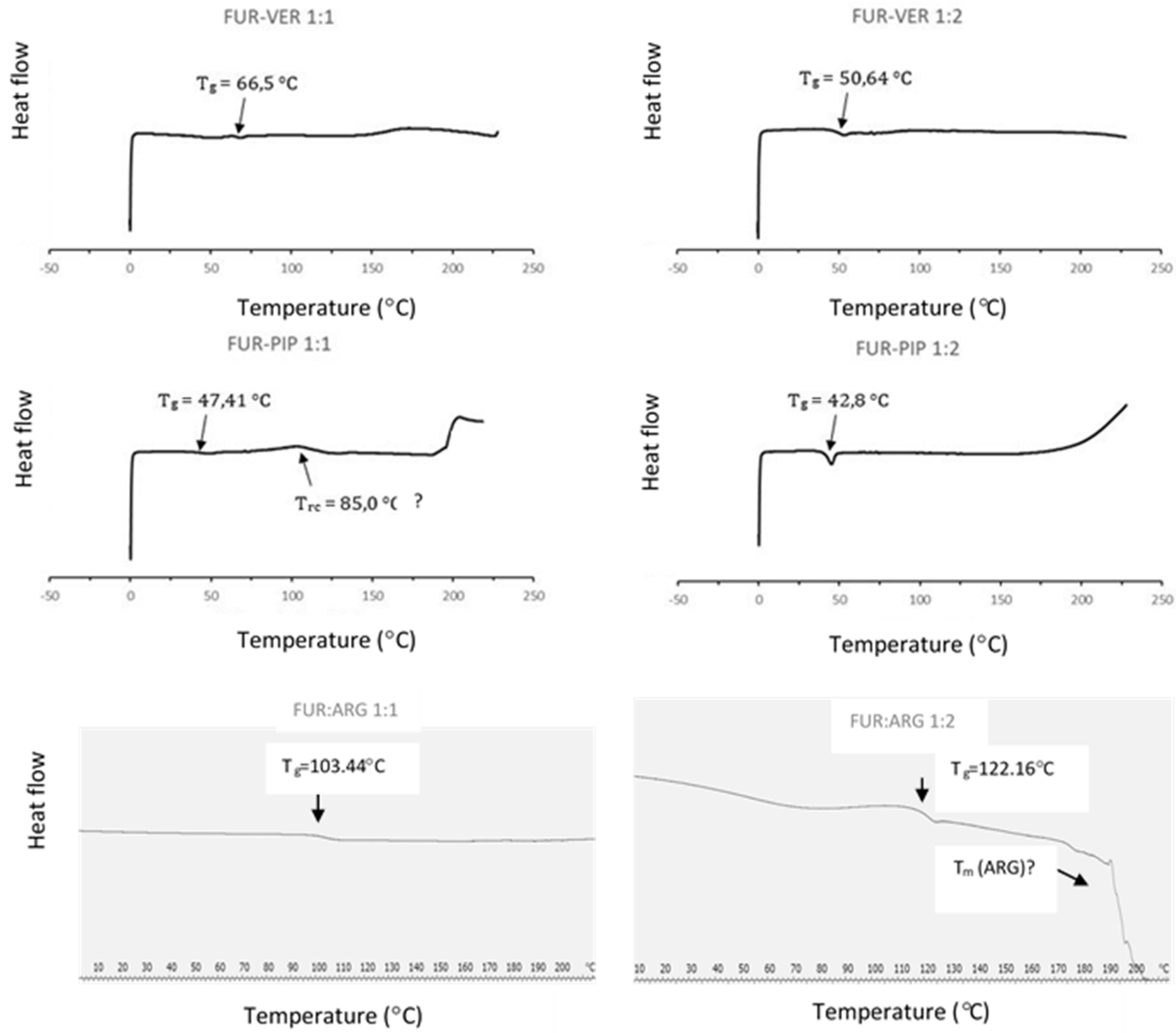

Figure S4. Example thermograms of the co-amorphous mixtures of furosemide (FUR), verapamil hydrochloride (VER), piperine (PIP) and arginine (ARG) at 1:1 and 1:2 molar ratios.

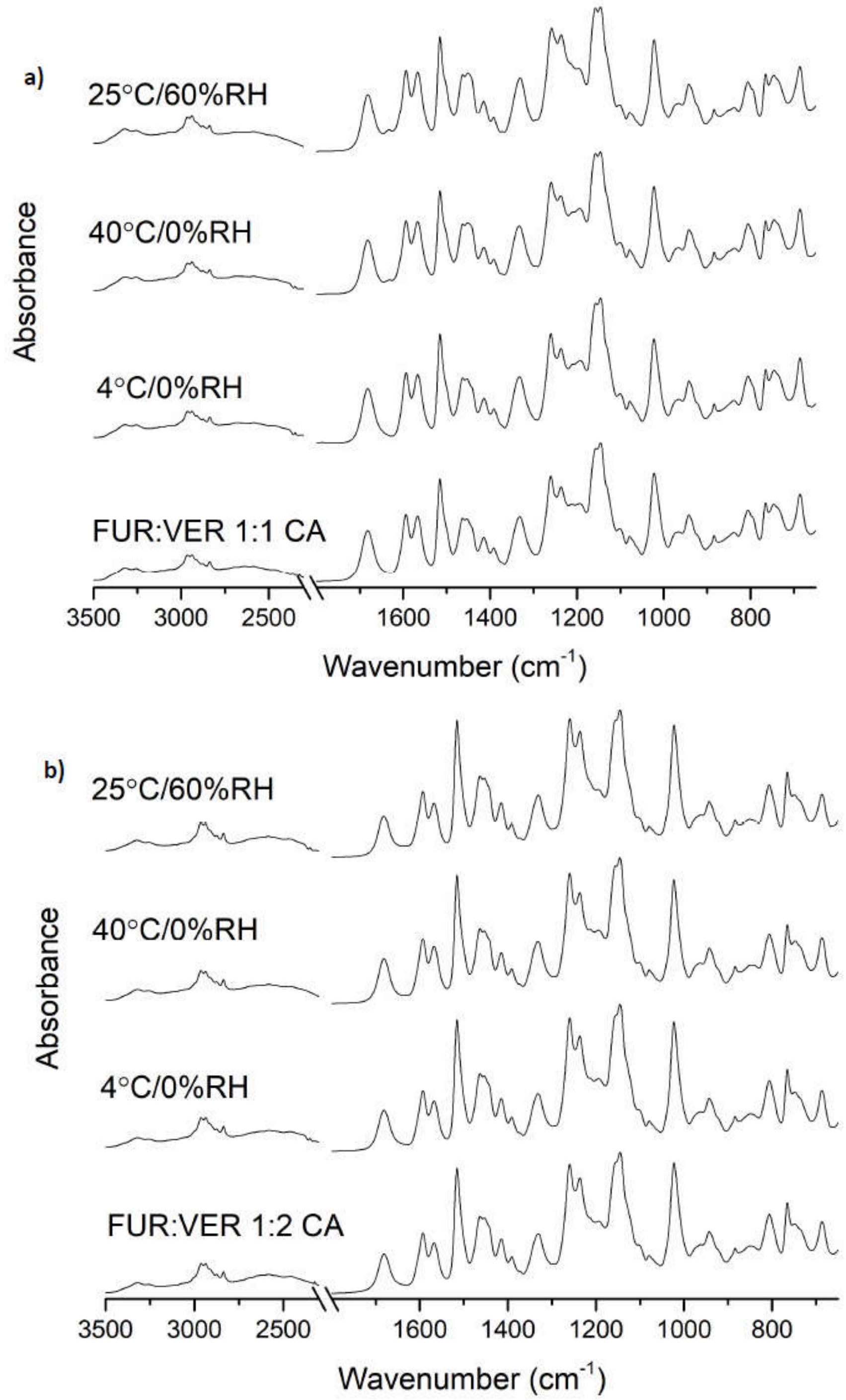

a)
25°C/60%RH
40°C/0%RH
4°C/0%RH
FUR:VER 1:1 CA
Absorbance
3500 3000 2500 1600 1400 1200 1000 800
Wavenumber (cm-1)
b)
25°C/60%RH
40°C/0%RH
4°C/0%RH
FUR:VER 1:2 CA
Absorbance
3500 3000 2500 1600 1400 1200 1000 800
Wavenumber (cm-1)

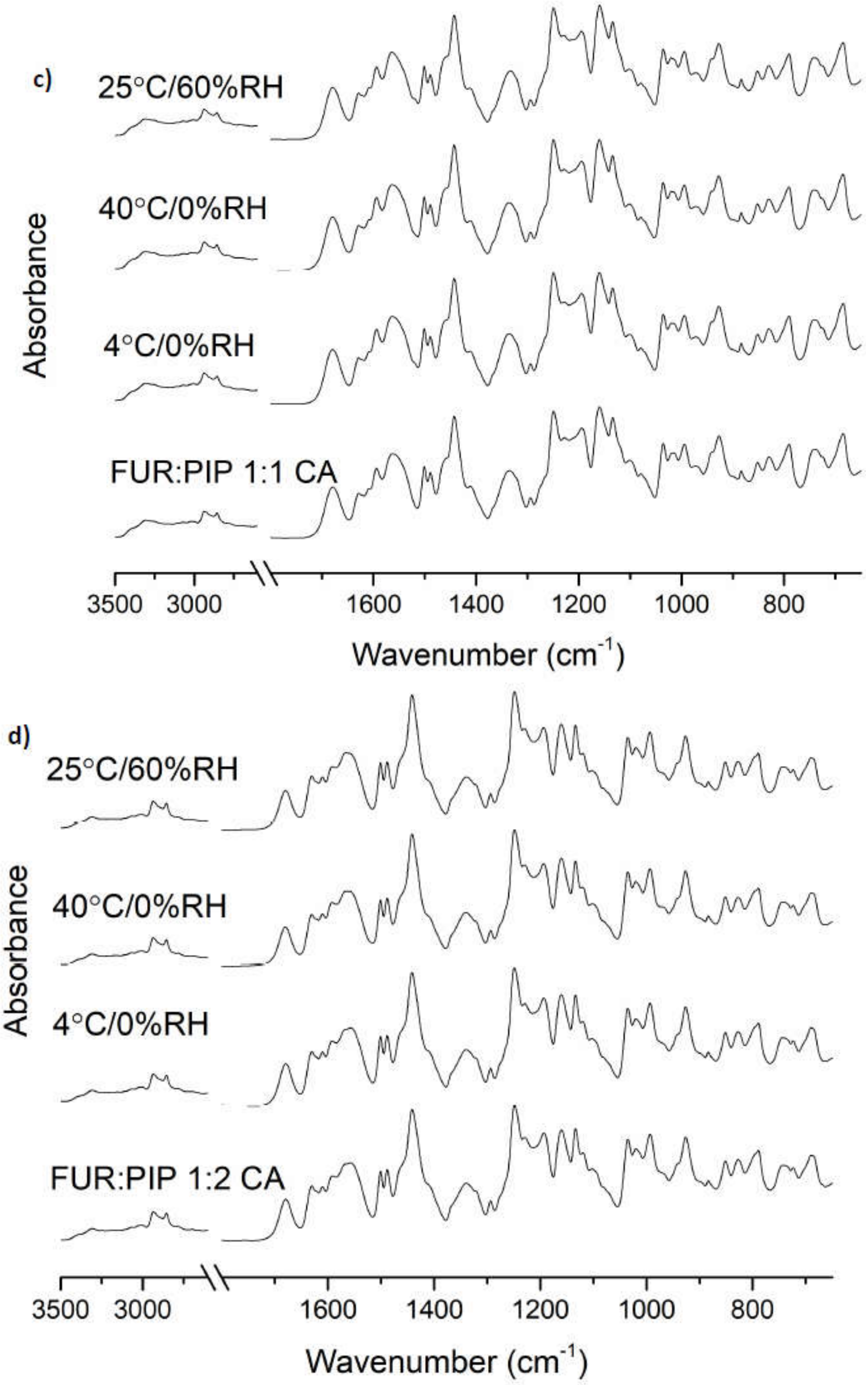
c)
25°C/60%RH
40°C/0%RH
4°C/0%RH
FUR:PIP 1:1 CA
Absorbance
3500 3000 1600 1400 1200 1000 800
Wavenumber (cm-1)
d)
25°C/60%RH
40°C/0%RH
4°C/0%RH
FUR:PIP 1:2 CA
Absorbance
3500 3000 1600 1400 1200 1000 800
Wavenumber (cm-1)

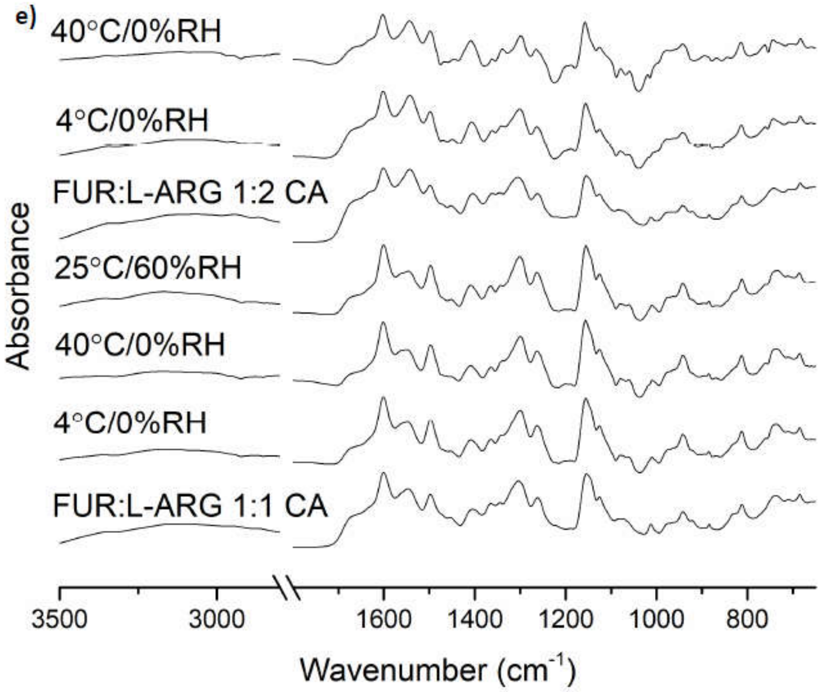

Figure S5. Fourier-transform infrared (FTIR) spectra of the co-amorphous materials after storage at 4°C/0% RH, 40°C/0% RH and 25°C/60% RH a) furosemide:verapamil hydrochloride (FUR:VER) 1:1 molar ratio at eight weeks; b) FUR:VER 1:2 molar ratio at eight weeks; c) furosemide:piperine (FUR:PIP) 1:1 molar ratio at eight weeks; d) FUR:PIP 1:2 molar ratio at eight weeks; e) furosemide:arginine FUR:ARG) 1:1 and 1:2 molar ratios at nine weeks.

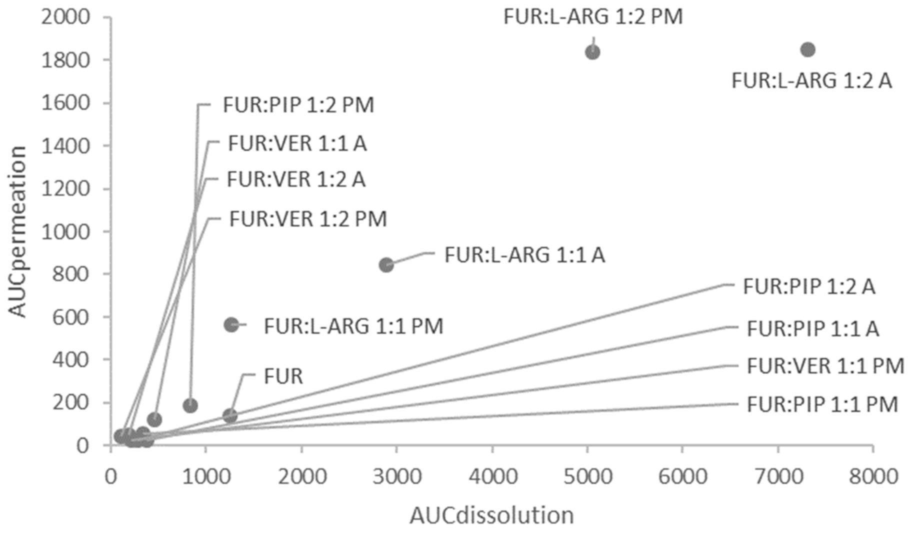

Figure S6. The correlation between the AUC values for dissolution and permeation.
